# High-Performance Polyolefin Material: Synthesis, Properties, and Application of Poly(4-Methyl-1-pentene)

**DOI:** 10.3390/ijms26020600

**Published:** 2025-01-12

**Authors:** Guangshui Tu, Handou Zheng, Jiahao Yang, Haotian Zhou, Chunyu Feng, Haiyang Gao

**Affiliations:** School of Materials Science and Engineering, PCFM Lab, GD HPPC Lab, Sun Yat-sen University, Guangzhou 510275, China; tugsh@mail2.sysu.edu.cn (G.T.); yangjh73@mail2.sysu.edu.cn (J.Y.); zhouht23@mail2.sysu.edu.cn (H.Z.); fengchy28@mail2.sysu.edu.cn (C.F.)

**Keywords:** poly(4-methyl-1-pentene), catalyst, polymerization, application, composite material

## Abstract

As a kind of high-performance thermoplastic crystalline resin, poly(4-methyl-1-pentene) (PMP) is characterized by its low density, low dielectric constant, exceptional mechanical and chemical properties, high transparency, and gas permeability. PMP has recently received more attention since COVID-19, because it is used as a hollow-fiber membrane for extracorporeal membrane oxygenation (ECMO) based on its high permeability and excellent biocompatibility. This review summarizes the chemical structure, synthesis, properties, and application of PMP. The advancements in catalyst systems for the catalytic synthesis of PMP, including Ziegler–Natta, metallocene, post-metallocene, and late-transition metal catalysts are emphasized. Furthermore, the molecular chain structure, helical conformation, and crystallization morphology of PMP, as well as its properties and applications, are also introduced in detail. Additionally, PMP composites and functional PMP materials are also described as promising and high-performance materials.

## 1. Introduction

Polyolefins have been playing prominent roles in the polymer industry as well as in our daily lives. At present, more than 300 different grades of polyolefins are commercially available for different applications [1]. Particularly, polyethylene (PE) and polypropylene (PP) are the most important materials produced by the petrochemical industry [2]. The properties of polyolefins are closely tied to the structures of their monomers. Different monomers generate polyolefins with distinct properties, resulting in varied application fields. In addition to PE and PP, there are a few high-performance polyolefin materials which also have irreplaceable application fields.

Poly(4-methyl-1-pentene) (PMP) is one of the high-performance thermoplastic crystalline resins, which is mainly prepared by isotactic coordination polymerization of 4-methyl-1-pentene (4MP) monomers. Natta first synthesized PMP using the Ziegler–Natta catalyst system in 1955. Subsequently, Imperial Chemical Industries (ICI) in the UK achieved semi-scale production of PMP in 1965. In 1973, this technology was sold to Mitsui Chemicals in Japan. In 1975, Mitsui Chemicals further developed ICI’s production technology for PMP and successfully initiated large-scale production of commercial PMP, which is marketed under the TPX™ brand. Currently, Mitsui Chemicals is the sole global supplier of PMP resin [3].

PMP is an isotactic polyolefin featuring isobutyl branches. The methyl side groups induce a helical structure that contributes to its unique properties. With a low density of 0.83 g/cm^3^, PMP stands out as the only semi-crystalline polymer with a crystal density lower than that of its amorphous phase [4], which is particularly advantageous in applications where weight savings are essential. Additionally, the presence of bulky side chains provides steric hindrance, which can inhibit the interaction of the polymer with various chemicals. PMP has a melting point of 230~240 °C, demonstrating excellent heat resistance. It also shows excellent mechanical properties, providing the processed materials with high strength levels. In addition to these features, PMP possesses several unique properties. As a high-performance, short-chain, branched isotactic polyolefin crystalline resin, PMP holds significant value across a diverse array of applications, including optical materials, electronic materials, and medical materials. The exceptional transparency of PMP makes it suitable for preparing food containers and experimental apparatus. Additionally, PMP’s outstanding electrical insulation properties facilitate the production of radio frequency insulators for fifth-generation mobile communication technology (5G) base stations. Notably, during the COVID-19 pandemic, extracorporeal membrane oxygenation (ECMO) has gained prominence as a critical intervention for treating severe respiratory failure in patients affected by the virus. PMP is used as a hollow-fiber membrane for ECMO due to its high permeability and excellent biocompatibility [5,6]. PMP membranes allow for efficient oxygen transfer while reducing the risk of thrombus formation and platelet activation. Moreover, the biocompatibility of PMP contributes to lower inflammatory responses, thereby making it safer for long-term applications [7]. Therefore, PMP has garnered significant attention as a special polyolefin resin. This review provides a comprehensive overview of the structure, catalyst systems, as well as the performance and applications of PMP. Moreover, PMP composite materials and functional PMP materials are also summarized.

## 2. Structure of PMP

**Figure 1:**
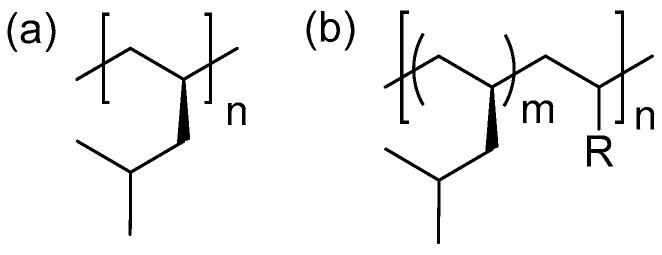
Polymer chain structures: (**a**) PMP; (**b**) poly(4MP-*co*-α-olefin).

PMP possesses an isotactic regular structure and exhibits a high melting point (up to 240 °C) along with high isotacticity ([mmmm] > 99%). However, these polymers exhibit certain brittleness in their material properties, which limits their widespread application. To enhance the toughness of PMP materials, the industry often introduces a small amount of α-olefin monomers and adjusts the comonomer insertion rate. The molecular structure of PMP is shown in Figure 1, where all isobutyl groups are positioned on one side of the chain in the two-dimensional Fischer projection. The polymer chains of PMP primarily exist in a linear configuration. The relatively regular arrangement of 4 MP monomers facilitates the stretching and movement of molecular chains, significantly influencing the material’s processing performance and deformation capabilities under specific conditions. For example, during the injection molding process, the linear molecular chains can flow and fill the mold to form products of various shapes.

Due to the presence of methyl side groups in the PMP polymer molecular chains, the molecular chain exhibits a secondary helical structure. These methyl side groups increase the steric hindrance of the molecular chains, causing them to twist in space. As a result, the arrangement of molecular chains is relatively loose, leading to a lower density within the crystalline region. The helical conformation has an important influence on the properties of the material. This loose arrangement of helical molecular chains enhances the permeation of gas molecules, making PMP an ideal material for the fabrication of gas separation membranes.

PMP, as a semi-crystalline resin with distinct crystalline regions, has molecular chains arranged in an orderly manner within the crystalline regions, forming a crystalline structure. In contrast to the crystallization behavior of highly isotactic PP commonly used in industry, PMP with a bulkier side group surprisingly exhibits a faster crystallization rate, because the significant steric hindrance effect allows longer and larger molecular chains to be incorporated into the crystalline region [8]. Currently, five crystalline modifications have been reported for PMP crystallized from solution [9]. Although all five crystal forms (Forms I, II, III, IV, and V) exhibit helical chains, the degree of helicity varies, leading to different helical conformations [4,10,11,12]. Form I, with a 7/2 helical conformation, is the most stable and can be obtained from melting or crystallization in high boiling point solvents [13,14]. In industrial applications, samples of Form I are commonly used [15]. The different crystalline forms of PMP exhibit varying degrees of helicity, resulting in differences in polymer density. The amorphous density is 0.838 g/cm^3^, while the most stable Form I has a lower crystal density than the amorphous density at room temperature [16], with an average density of 0.830 g/cm^3^. The distinctive density attributes of PMP also account for its rapid crystallization. Nucleation plays a pivotal role in the crystallization process of PMP, wherein the mobility of molecular chains dictates the rate of nucleus formation and growth. Nucleating agents possess favorable dispersibility and stability, endowing semi-crystalline polymers with outstanding mechanical and optical properties [17]. As a highly isotactic PMP polymer, when specific nucleating agents are added during the polymer crystallization process, they can not only enhance the crystallization rate and crystallinity but also improve its performance [18]. Additionally, the crystalline phase of PMP is permeable, facilitating the permeation of gas molecules. Consequently, PMP can be used to prepare gas separation membranes [19] and extracorporeal membrane oxygenation [20], both of which play a significant role in the treatment of patients with severe COVID-19. Furthermore, the presence of isobutyl branches in the molecular chain skeleton of PMP results in the density and refractive index of its crystalline and amorphous regions being essentially the same. This ultimately results in PMP having high transparency properties with transparency exceeding 90% and haze below 3% [21].

## 3. Catalytic System of PMP

PMP resin is primarily obtained through the coordination polymerization of the 4 MP monomers. The choice of catalyst is critical in influencing the isotacticity of the resulting polymer. Currently, four main catalyst systems are commonly employed in the preparation of PMP, namely Ziegler–Natta catalyst, metallocene catalyst, post-metallocene catalyst, and late-transition metal catalyst.

### 3.1. Ziegler–Natta Catalyst

Isotactic PMP was first produced in 1955 by Natta and coworkers using Z-N catalysts, but the activity and isotacticity are low [22]. Following this, extensive research efforts were devoted to enhancing both the catalytic activity and the isotacticity of Z-N catalysts. In order to obtain high yields of crystalline polymers without much atactic byproduct, highly isotactic catalysts must be used. The early use of homogeneous Z-N catalysts for the polymerization of 4 MP exhibited not only poor catalytic activity and isotacticity but also several problems during the polymerization process. For example, the polymer morphology is poor, and the reactor is prone to scaling, making it difficult to separate products and catalysts. Subsequent research on polymerization has primarily focused on heterogeneous catalysts. MgCl_2_ supported Z-N catalysts can efficiently catalyze the polymerization of 4 MP. MgCl_2_-supported Ti catalysts have shown high polymerization activity and fair-to-good stereospecificity for the isotactic polymerization of 4 MP [23,24,25]. Ciardelli used supported bimetallic (Ti and Hf) Z-N catalysts to catalyze the homopolymerization of 4 MP [26]; the isotacticity and viscosity increase with the increase in the Hf/Ti ratio, while the specific activity is almost unaffected, and isotacticity increases up to 93.7%.

By adding an electron donor (ED) to the Z-N catalyst, the isotacticity and crystallinity of the polymer are significantly improved, and the molecular weight and molecular weight distribution can be better controlled. EDs are often added to make more stereospecific ternary catalyst systems. The donor compound is believed to act as a mild poison, preferentially poisoning the more active but less stereospecific sites. For the Z-N catalyst system, the commonly used EDs are esters, ketones, ethers, amines, and organosilanes. Kleiner carefully studied the effects of different external donors on the activity and stereospecificity of the MgCl_2_/TiCl_4_/Al*^i^*Bu_3_ catalytic system in the bulk polymerization of 4 MP [27]. Different silane compounds of the structure R_n_Si(OR’)_4-n_ (n = 1–3, R = alkyl/phenyl, R’ = alkyl) were used as external donors; the activity and stereospecificity of the catalytic system simultaneously decrease as the size of the alkyl group gets larger, and the most effective alkylalkoxysilanes are methyl and methoxy derivatives, with moderate catalytic activity (4.5 × 10^3^ g_pmp_·g_cat_^−1^) and high isotacticity (up to 98%). Mitsui Chemicals studied the effect of donor compounds on the activity and stereospecificity of MgCl_2_/TiCl_4_/diisobutyl phthalate [25], which not only has high activity (up to 2.56 × 10^7^ g_pmp_·mol_Ti_^−1^) but also high isotacticity (up to 98.2%). The stereoregularity of PMP decreases with increasing polymerization temperature due to the isotactic and atactic polymerization sites having different activation energies [28].

Currently, isotactic PMPs synthesized using Z-N catalyst systems are typically copolymers with varying amounts of α-olefin comonomers. Random copolymers of 4 MP, with ca. 2~8 wt% of C_6_–C_12_ linear α-olefins, are important commercially because of their good transparency and robust processing behavior [3]. Ciardelli used the MgCl_2_-Hf/Ti catalytic system to catalyze the copolymerization of 4 MP with monoalkenes such as propylene, 1-butene, and 1-hexene, showing ta higher molecular weight (intrinsic viscosity (η) up to 9.5 dl/g for 4 MP-hexene copolymer) with the Hf-supported catalysts [26]. Campbell reported the copolymerization of 4 MP with 1-pentene, 1-hexene, 1-octene, and 1-octadecene, resulting in crystalline copolymers with high melting, excellent solubility in solvents such as cyclohexane or chloroform, and the ability to readily form films and fibers [29]. Jones further investigated the crystallization behavior of isotactic copolymers of 4 MP with linear α-olefin comonomers (1-pentene, 1-hexene, 1-octene, 1-decene, and 1-octadecene) over a wide range of copolymer composition [30]. With the increase in side chain length and comonomer content, the degree of crystallinity of PMP decreases; 4 MP-pentene copolymers with a pentene content of 11% has high crystallinity (up to 59%).

Mizuno studied the structure and crystallinity of the copolymer of 4 MP with a small amount of 1,5-hexadiene, finding that the resulting copolymers contain two rigid cyclic units, 4 MP and 1-methyl-3-cyclopentenyl [31]. The introduction of the 1-methyl-3-cyclopentenyl unit into the 4 MP sequences reduces the crystallinity of the polymer. Kisssin also investigated the structure of copolymers of 4 MP-styrene using FTIR spectroscopy but obtained a random copolymer [32]. Therefore, the copolymerization of 4 MP with a small amount of linear α-olefin comonomers is the most effective method to improve the brittleness of PMP.

### 3.2. Metallocene Catalyst

Unlike Z-N catalysts with multiple active centers, metallocene catalysts with a single active center result in a narrower molecular weight distribution for polymers. Mitsui Chemicals also mentioned in their patent that the metallocene catalyst system can efficiently catalyze the polymerization of 4 MP [33]. The structure and symmetry of metallocene catalysts are important parameters that influence the isotacticity of the resulting polymers [34].

**Figure 2:**
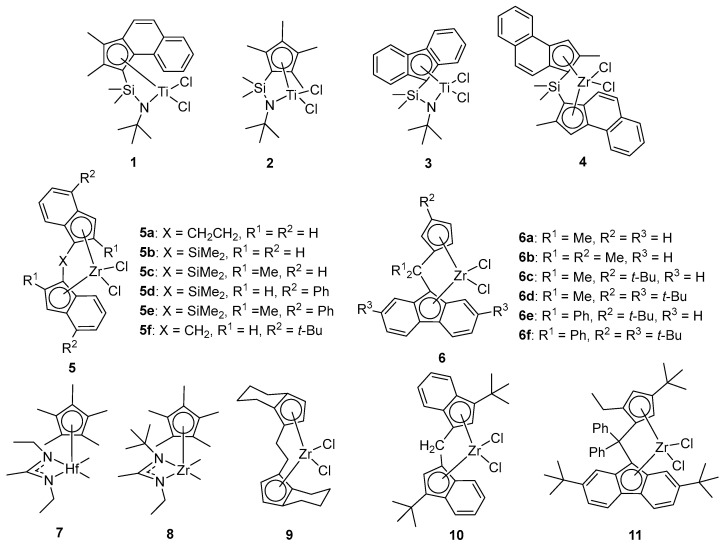
Metallocene catalysts for 4 MP polymerization.

Xu and coworkers catalyzed the polymerization of 4 MP using a series of constrained-geometry complexes (CGCs) and a bridged metallocene catalyst [35]. The structure of the catalysts is shown in Figure 2. It was found that CGCs (**1**–**3**) and the bridged catalyst (**4**) exhibit high catalytic activity (~10^6^ g·mol^−1^·h^−1^) and narrow molecular weight distributions (MWDs) (<3). The larger the steric hindrance of substituents in CGCs, the higher the molecular weight of PMP, but the isotacticity is poor (<40%), while the activity of 4 was higher (5 × 10^6^ g·mol^−1^·h^−1^), and the isotacticity of PMP was also increased to 94%. Duchateau used a series of *C*_2_-, *C_s_*-, and *C*_1_- symmetric zirconocenes to catalyze 4 MP polymerization [36]. The polymerization activities for *C*_2_-symmetric zirconocenes at varying temperatures are **5a** > **5d** >> **5b** > **5c** > **5e**. Except for catalyst **5c**, the molecular weights of the PMPs using these catalysts are relatively low (M_w_ ≤ 42.2 × 10^4^ g·mol^−1^), and all PMPs exhibit relative narrow MWDs (≤3.3) and a high melting temperature (T_m_ ≥ 200 °C). Unlike the *C*_2_- symmetric zirconocenes, for *C*_1_- and *C_s_*- symmetric zirconocenes catalysts **6a**–**6f**, the substituents on the bridge, the cyclopentadienyl (Cp) and fluorenyl (Flu) ligands, have a significant impact on the polymerization of 4 MP. The Cp tert-butyl substituent enhanced the catalyst activity, polymer molecular weight, and regioregularity at low temperatures, while the regioregularity is lower at higher temperatures. Phenyl substituents on the bridge have a limited effect on the polymer molecular weights. The molecular weights of the polymers were considerably higher than those of the PMPs obtained with **5a**–**5e**, and the activities of these catalysts are also very high compared to the corresponding *C*_2_- symmetric catalysts. The *C*_1_- symmetric catalyst **6f** has the highest activity (up to 4.38 × 10^6^ g·mol^−1^·h^−1^), obtaining PMPs with high isotacticity, high M_w_ (up to 1.7 × 10^5^ g·mol^−1^), narrow MWDs (≤2.2), and high T_m_ (up to 238 °C).

Longo used the *C*_2_- symmetric zirconocene catalyst **5f**/MAO system to catalyze the polymerization of 4 MP with moderate polymerization activity (4.3 × 10^4^ g·mol^−1^·h^−1^) and obtained isotactic PMP with a relatively high molecular weight (M_w_ = 5.08 × 10^4^ g·mol^−1^) and narrow MWD (2.1) [37]. Sita and coworkers studied the stereomodulation of PMP tacticity to provide new fundamental forms that enable the adjustment of thermal phase transitions (T_g_ and T_m_) over a wide range [34]. Recently, they also demonstrated that PMP with an adjustable range of viscoelastic properties can be achieved in “one-pot” fashion by controlling the ratio of hafnium **7** and zirconium **8** catalysts through living coordinative chain transfer polymerization, and the elongation at break of PMP is up to 350% [38].

Similarly, metallocene catalysts can also catalyze the copolymerization of 4 MP with ethylene, propylene, and 1-pentene. Sacchi and coworkers synthesized 4 MP/E random copolymers using **CGC 2** [39], employing the *C*_2_- symmetric metallocene catalysts **9**, **5e**, and **5f** to produce 4 MP/E copolymers with high stereoregularity and regioselectivity [40,41,42]. Additionally, Galimberti synthesized 4 MP/E blocky copolymers by controlling the 4 MP/E feed ratio in the liquid phase using catalysts **5a**, **9**, and **10** [43]. Sacchi and coworkers also synthesized 4 MP/P random copolymers utilizing metallocene catalysts **4**, **9**, and **10** [44,45]. Koval’chuk [46] and Okamoto [47] synthesized 4 MP/P random copolymers using metallocene catalyst **5e** and **11,** respectively, which exhibited excellent rubber elasticity. Furthermore, both random and block copolymers based on 4 MP/1-pentene have been synthesized using zirconium catalyst **8** [48].

### 3.3. Post-Metallocene Catalyst

**Figure 3:**
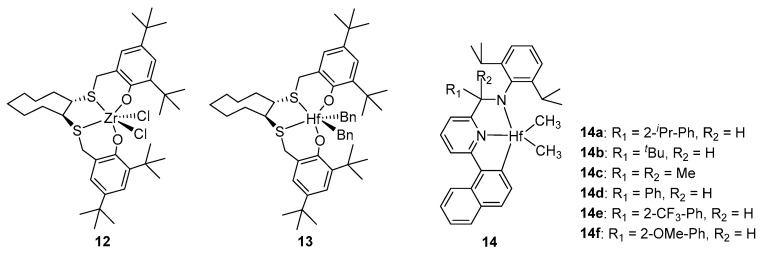
Post-metallocene catalysts for 4 MP polymerization.

Post-metallocene catalysts have been the focus of extensive research due to their ability to produce highly isotactic polymers during the catalytic polymerization of α-olefins. Among these catalysts, post-metallocene hafnium catalysts exhibit particularly remarkable performance in catalyzing the polymerization of 4 MP. Ishii reported [OSSO]-type catalysts zirconium **12** [49] and hafnium **13** [50] (Figure 3) catalyzing the polymerization of 4 MP. The activity of the zirconium catalyst is higher (10^6^ g·mol^−1^·h^−1^), and both catalysts yield PMP materials with narrow MWDs (<2.1) and high isotacticity ([mmmm] > 95%).

The pridylamido hafnium catalyst is a high-performance post-metallocene catalyst newly developed by Symyx Technologies and Dow Chemicals [51], which has high activity, excellent isotacticity, high molecular weight, and a narrow molecular weight distribution in catalyzing α-olefin polymerization. Our group conducted a detailed study on the homopolymerization of 4 MP catalyzed by pridylamido hafnium catalysts **14a**–**14c** (Figure 3) with three different structures [52]. With the increase in temperature, the polymerization activity first increases and then decreases, and the activity was the highest (up to 10^7^ g·mol^−1^·h^−1^) at 40 °C. Moreover, regardless of the bridgehead substituent and polymerization temperature, highly isotactic PMPs ([mmmm] > 99%) with high melting temperatures (T_m_ = 229–240 °C) were produced. We utilized the classical pridylamido hafnium catalyst **14a** to catalyze the copolymerization of 4 MP with 1-hexene and prepared a random copolymer [53]. Li and coworkers also synthesized a random copolymer of 4 MP and 1-butene using the hafnium catalyst **14a** [54]. Several pridylamido hafnium catalysts (**14a** and **14d**–**14f**) with different electronic effects were synthesized by Jian and used to catalyze the polymerization of 4 MP [55]. No matter how the bridgehead substituent of hafnium catalyst changes, the obtained PMP exhibits high isotacticity ([mmmm] > 99%) and melting temperatures (T_m_ > 232 °C). Recently, Stefano synthesized 4 MP/1,5-hexadiene isotactic copolymers incorporating methylene-1,3-cyclopentane (MCP) cyclic co-units using hafnium catalyst **14a**. All crystalline isotactic copolymers exhibit high melting temperatures (T_m_ > 120 °C), and a controlled glass transition temperature close to room temperature (28–30 °C). Incorporating MCP units into PMP chains produces an improvement in flexibility and allows for the tailoring of deformability while retaining high mechanical resistance and transparency of the homopolymer [56].

### 3.4. Late-Transition Metal Catalyst

**Figure 4:**
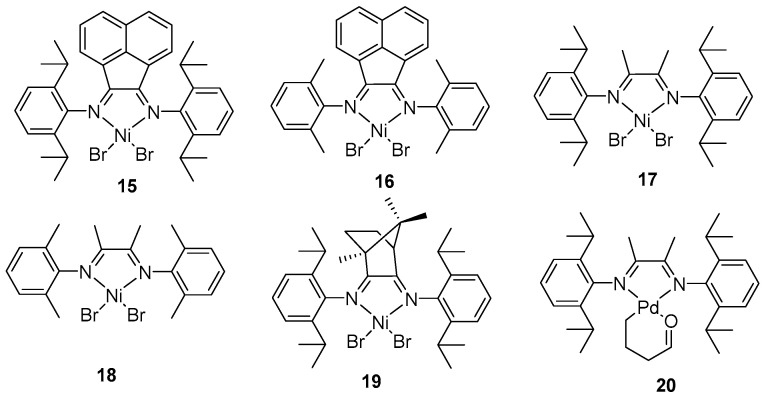
α-Diimine nickel and palladium catalysts for 4 MP polymerization.

A typical late-transition metal catalyst system is the α-diimine Ni and Pd catalysts, which efficiently catalyze the polymerization of 4 MP with high activity. Furthermore, the introduction of bulky steric substituents into the ligand structure can effectively inhibit chain transfer reactions [57,58,59,60], thereby facilitating the production of high-molecular-weight polyolefins. Our group has reported a series of α-diimine nickel catalysts **15**–**19** and a palladium catalyst **20** (Figure 4) for the polymerization of 4 MP [61,62,63,64], all of which demonstrate high activity (10^5^ g·mol^−1^·h^−1^) and yield high-molecular-weight (10^5^ g·mol^−1^) PMP with narrow MWDs (<2). Ricci also reported the copolymerization of ethylene with 4 MP using α-diimine nickel catalyst **17** resulting in random copolymers [65,66]. Due to the unique chain-walking mechanism present in the catalytic polymerization processes of these late-transition metal catalysts, branched products are formed, leading to PMPs that are amorphous elastomers with a low glass transition temperature.

In summary, four types of catalysts are capable of catalyzing the polymerization of 4 MP. Z-N catalysts have been widely utilized in polyolefin polymerization, exhibiting moderate catalytic activity to produce broad molecular weight distribution polymers for the polymerization of 4 MP. Metallocene catalysts possess a single active center, thereby producing normally distributed polymers. Metallocene catalyst structure and symmetry have a great impact on the isotacticity of PMP polymers, and the resultant PMP polymers often have relatively low isotacticity and molecular weight. Post-metallocene catalysts display very high catalytic activity to afford highly isotactic PMP with normal distributions, but their industry application needs to solve catalyst immobilization. Late-transition metal catalysts show very low isoselectivity and commonly produce amorphous polyolefins with complex branching structure because of chain walking.

## 4. Properties and Applications of PMP

Compared to traditional polyolefins, PMP possesses unique chain and helical structures. These structures not only endow PMP with excellent mechanical properties but also bestow unique characteristics, such as a high melting point, low density, low dielectric constant, high transparency, and high gas permeability. Therefore, PMP plays an irreplaceable role in certain special application fields.

### 4.1. Excellent Heat Resistance

The bulky side chain configuration of PMP hinders thermal movement between molecules, consequently leading to outstanding heat resistance, with a melting point ranging from 230 to 240 °C. Compared to commonly used transparent polyolefin materials (PE, PP, PS, etc.), PMP also boasts a higher vicat softening point, ranging from 160 to 178 °C. Additionally, PMP has a relatively high heat distortion temperature, which can reach 110~130 °C under a stress of 0.45 MPa. The excellent heat resistance allows PMP to maintain good flexibility, fracture elongation, and impact strength at elevated temperatures, making it suitable for various applications, including automotive parts, high-temperature resistant cling film, baking trays, and microwave-safe containers. For example, Mitsui Chemicals has developed a PMP food preservation film with excellent temperature tolerance. This film can withstand temperatures of up to 180 °C and does not become brittle at −30 °C. Moreover, the density of PMP is only 0.83 g/cm^3^, the lowest among all thermoplastic materials, which can significantly reduce production costs in industrial applications.

### 4.2. Low Dielectric Property

PMP has a non-polar structure, and its dielectric properties are almost equivalent to those of fluorine polymers and the best cable-grade PE (Table 1). Its dielectric constant (ε = 2.068 F/m) is the smallest of all synthetic plastics, and the dielectric dissipation (12 GHz, tan δ = 0.0008) is also very low, and it remains essentially constant over wide temperature and frequency ranges [67]. Therefore, PMP is highly suitable for applications in high-frequency fields, such as printed circuit boards, 5G base station accessories, insulators for high-voltage power lines, and other high-frequency electrical appliances. Due to its negligible moisture absorption, PMP preserves these electrical characteristics under adverse conditions [3]. In addition, PMP is also used as a dielectric capacitor film for high-temperature energy storage applications due to its low dielectric properties [68].

### 4.3. High Transparency

**Figure 5:**
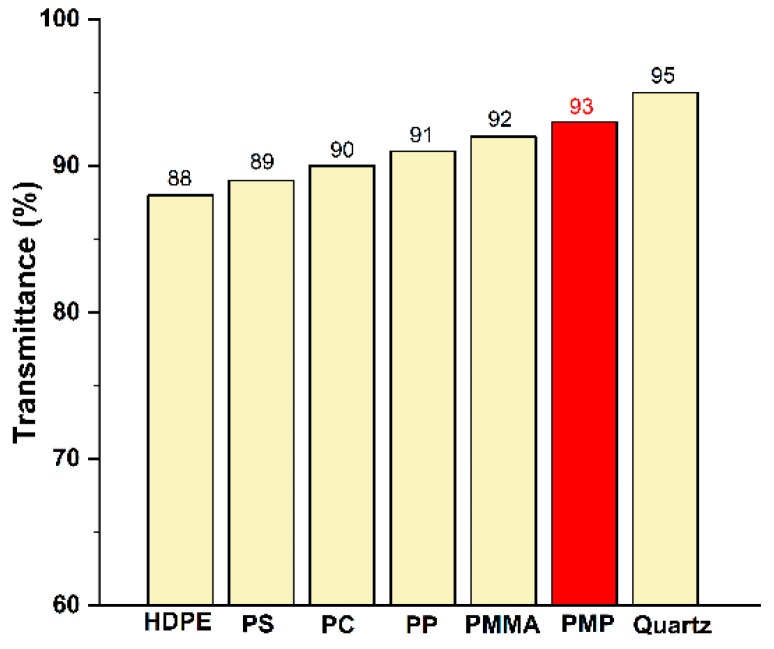
Transmittance of different polymeric materials.

The unique helical molecular chain structure of PMP significantly reduces the optical anisotropy of its molecules, leading to a density of the crystalline and amorphous regions that is quite similar. This characteristic contributes to PMP’s impressive optical isotropy and transparency, with a haze value of less than 5%. Commercial PMP resins typically achieve optical transmittance in the range of 90–93%, with transmittance that surpasses that of most common polymers (Figure 5). PMP stands out as the only crystalline polymer among high-transparency resins. This excellent optical performance makes PMP highly suitable for various applications, such as lighting fixtures, food containers, animal feeding enclosures, laboratory equipment, and other sectors where clarity and transparency are essential. Moreover, compared to other transparent materials, PMP holds a distinct advantage. For instance, while materials like polycarbonate (PC) and PMMA are commonly used for their clarity, they can exhibit higher levels of optical distortion and lower UV resistance. PMP’s low haze and superior UV transmission make it especially attractive for applications requiring prolonged exposure to sunlight without significant yellowing or degradation, and its unique properties position it as a formidable contender in the transparent resin market.

### 4.4. Excellent Chemical Resistance

Due to its stable C-C bonds, PMP has better chemical resistance compared to PC, polyamide (PA), and acrylic polymer (Table 2). PMP shows excellent resistance, particularly against acids, alkalis, alcohol, and ketones. Some aromatic and halogenated hydrocarbon solvents can reduce the tolerance of PMP but only cause slight swelling and some strength loss. Therefore, PMP is more suitable for various applications that require chemical resistance, such as cosmetic container caps and tubes, experimental apparatus, and analytical cells.

### 4.5. Low Surface Tension

**Figure 6:**
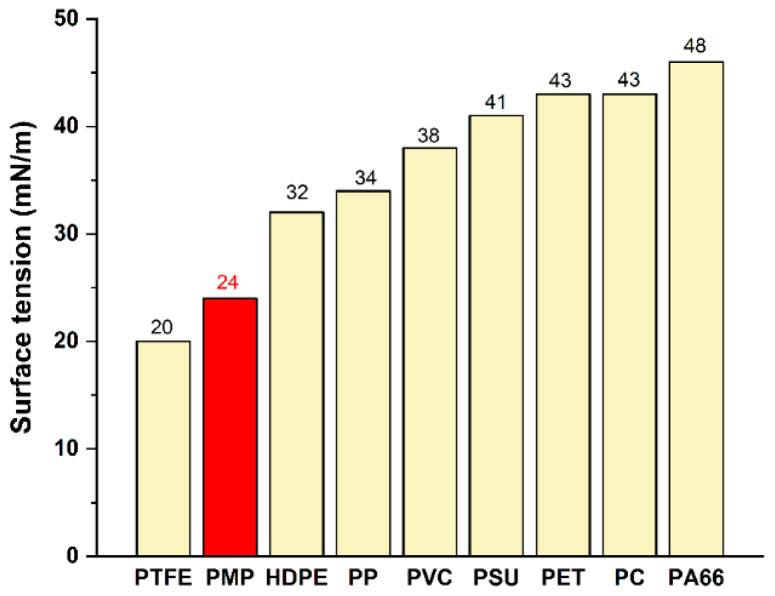
Surface tension of different polymers.

The surface tension (24 mN/m) of PMP is very low, which makes it easy to peel off, and it has lower peel ability than most traditional polyolefins, being slightly higher than polytetrafluoroethylene (Figure 6). Extremely low surface tension makes it difficult to adsorb water or oil. In addition, PMP exhibits excellent releasability against various materials in the hardening process of thermosetting resins (urethane, epoxy, etc.). It also exhibits incompatibility with thermoplastic resins (PET, PP, etc.) and can be used to create porous structures in PET or films. Therefore, PMP has been widely used in fields such as food containers, FPC release films, rubber hoses, release paper for synthetic leather, resin modification, and LED mold strips.

### 4.6. Excellent Gas Permeability

The large helical molecular chain structure of PMP results in loose intermolecular stacking, which contributes to its excellent gas permeability [69,70]. Studies have demonstrated that the O_2_ permeability of PMP is 12 times that of PP and 18 times that of high-density polyethylene (HDPE) at 23 °C (Table 3). This high permeability is a critical advantage for its application in gas separation technologies, particularly in medical devices such as ECMO. PMP’s unique molecular structure facilitates the separation of N_2_/O_2_/CO_2_ due to its differential permeation characteristics. The ability of PMP membranes to preferentially allow O_2_ to permeate while restricting N_2_ and CO_2_ makes it an ideal candidate for ECMO systems and other gas separation tasks in medical applications [71]. Furthermore, the selectivity of PMP membranes towards O_2_ over N_2_ and CO_2_ enhances the efficiency of the gas exchange process, ultimately leading to better patient outcomes in critical care settings. The selective gas permeation plays a significant role in optimizing the performance of ECMO devices, allowing for the effective management of hypoxemia in patients with severe respiratory failure [72].

In addition to the medicine field, the excellent permeability of PMP is also used in renewable energy. The crystalline phase of PMP has gas selective permeability characteristics, with high selectivity for the separation of C_1_-C_4_ alkane [19,73], which is of great significance for the regulation of energy products in biomass processing and other renewable energy-related gas separation processes. PMP is also employed as part of an ion exchange system for the recovery of ammonia from wastewater [74]. The high gas selectivity of PMP helps to improve energy utilization efficiency and resource recovery utilization in renewable energy production processes.

### 4.7. Good Biocompatibility

PMP, as a high-performance polyolefin material, exhibits low cytotoxicity and does not release harmful substances. When cultured with cells, it maintains normal cell morphology and viability over extended periods, thereby demonstrating excellent biocompatibility. Therefore, PMP materials have a wide range of applications in the medical and health field.

For example, PMP asymmetric membranes can be used to make subcutaneous syringes, blood collection and transfusion equipment, and pacemaker components [3]. For implantable medical applications, the biocompatibility of PMP is of utmost importance. It can be used in the fabrication of certain types of implants, such as small-scale implants for local drug delivery systems and biodegradable drug release devices [75,76]. In terms of orthopedic implants, although PMP is not as widely used as traditional materials such as titanium alloys, its lightweight nature and good biocompatibility provide advantages. In some minimally invasive surgeries, lighter implants can reduce patient trauma during implantation and recovery processes.

PMP is also an excellent choice for microfluidic applications. Its remarkable plasticity allows for the precise fabrication of complex microchannel structures, making it ideal for the production of microfluidic chips [77]. These chips play a crucial role in real-time diagnostics, enabling the rapid analysis of biological samples.

Moreover, PMP, a typical non-polar hydrophobic material, can cause coagulation and thrombosis problems when in contact with blood. In order to improve the blood compatibility of PMP membranes, coating technology is employed to modify the prepared membrane substrate. For instance, covalently grafting heparin onto the surface of PMP membranes has been shown to significantly improve hemocompatibility [78,79]. Additionally, catalytic coatings that generate nitric oxide on the membrane surface enhance blood anticoagulant properties [79]. Other modifications include grafting the enzyme carbonic anhydrase onto the membrane surface to improve the carbon dioxide removal rate [80] and applying titanium dioxide coatings to enhance the anti-serum leakage function of PMP membranes [81]. Coupling zwitterionic sulfobetaine block copolymers to PMP hollow-fiber membrane surfaces has also proven effective in reducing thrombus formation in respiratory assist devices [82]. Furthermore, some researchers have adopted a biomimetic approach by cultivating and growing a layer of endothelial cells on the surface of the PMP membrane. This method achieves biocompatibility that closely resembles that of natural blood vessels [71,83].

## 5. PMP Composite Material

### 5.1. Blending of PMP and Polymers

The blending of PMP with other polymers not only presents an effective strategy for cost reduction but also significantly enhances their mechanical properties. In the following content, we will conduct an in-depth discussion on the blending research of PMP with PE, PP, poly(1-butene) (PB), and polycarbonate (PC).

PE is characterized by its flexibility and ductility. When it is blended with the relatively rigid PMP, the cost of PMP can be reduced, while also enhancing the processability of the resulting materials. Li and coworkers obtained a PMP/ultra-high-molecular-weight polyethylene (PMP/UHMWPE) blend film through sequential biaxial stretching for Li-ion batteries [84]. PMP has high gas permeability, its incorporation results in high porosity and wettability, significantly increasing air permeability, electrolyte uptake, and ionic conductivity. The relationship of processing–structure–property of PMP/low-density polyethylene (LDPE) blends has also been studied [85]. Gokkurt prepared a series of PMP/LDPE blend films using a twin-screw extruder melt composite method. The results indicated that the incorporation of PMP enhanced the mechanical properties as well as the O_2_ and CO_2_ permeability of LDPE. When the PMP content is low, the blend film does not exhibit the specified oriented lamellar structure. As the content of PMP increases (≥50%), the film exhibits homogeneously directed lamellar structures in the flow direction. Compared with the traditional flexible packaging material LDPE, the blend film exhibited better performance and can be used as a highly permeable packaging material to extend the shelf life of fresh-cut fruits and vegetables [86].

The blending of PMP and PP combines the advantages of both. The gas permeability of the composite material will be improved, and the mechanical strength will also be improved to some extent. Bu and coworkers conducted research on PMP/PP blends and found that they indeed have good compatibility [87]. Jiang and coworkers used thermally induced phase separation to blend PP as a high-rigidity reinforcing agent with PMP and prepared polymer alloy oxygenated membranes that are easy to process and can resist plasma leakage for a long period of time [6]. Moreover, Givens prepared PMP/PB blend fiber membranes via electrospinning technology, which maintain the excellent physical properties of both while improving their flexibility [88]. Ilyin fabricated a series of microfiltration membranes from PMP/polyisobutylene(PIB) blends, which can be considered as a prospective system for the fabrication of microfiltration membranes by melting processing that might include 3D printing techniques [89,90]. For the PMP/PC blend, both the optical and mechanical properties are improved. The blending of PMP and PC can improve the impact resistance and weather resistance of composite materials [91].

### 5.2. PMP Nanocomposite Materials

PMP can not only be blended with polymers to improve performance but also combined with inorganic nanomaterials such as graphene oxide (GO-ODA), silica (SiO_2_), titanium dioxide (TiO_2_), clay, and fiber materials to significantly enhance the mechanical properties and thermal stability of PMP.

Yin and coworkers blended different amounts of alkylated GO-ODA with PMP through a solution method to obtain nanocomposites. GO-ODA has no effect on the crystallinity of PMP but changes the crystal structure and improves the mechanical properties of the composite material [92,93]. Dorigato prepared PMP/silica nanocomposites by melt compounding 2 vol% fumed silica nanoparticles of various surface areas. The addition of nano silica fillers has almost no effect on the transparency of PMP, but its mechanical properties (especially the creep stability) have been improved, and the storage modulus has also been significantly increased above the glass transition temperature of the polymer matrix [94]. Ahmad continued to fabricate mixed-matrix membranes (MMMs) of PMP with nano fumed silica particles for CO_2_/N_2_ gas separation [95]. By using the inorganic material TiO_2_ as a reinforcing agent and controlling the solubility of TiO_2_ filler and PMP in different solvents, high-performance PMP/TiO_2_ composite films can be synthesized [96]. Park also fabricated PMP/TiO_2_ nanocomposites by solution mixing poly(ethylene-co-vinyl alcohol) (EVOH)-coated TiO_2_ (c-TiO_2_) nanoparticles [97]. Introducing a small amount of c-TiO_2_ can significantly improve the tensile strength of the PMP matrix, and compared with the pristine PMP, the insulation resistance of the nanocomposites is higher, and the electrical insulation performance has been greatly improved. Nanocomposites of PMP/clay were successfully prepared using a melt intercalation technique, resulting in a new type of PMP composite material with a lower crystallinity and thermal expansion coefficient, higher thermal stability, and higher dynamic storage modulus at high temperatures [98,99].

By blending PMP with glass fiber, a lightweight and inexpensive reinforced PMP material with higher heat resistance and mechanical properties was obtained. In addition, by blending PMP with natural fibers, green and environmentally friendly composite materials can be prepared. Khatri fabricated PMP/co-electrospun cellulose (CA) nanofibers by electrospinning technology followed by deacetylation, and the tensile strength of the nanofibers was improved [100]. When the mixing ratio of CA and PMP is 1:1, it has the highest tensile strength (6.9 MP) and Young’s modulus (195 MPa), and nanofiber membranes with improved mechanical strength can be used as a value additive for cellulose-based membranes.

## 6. Functional PMP

**Figure 7:**
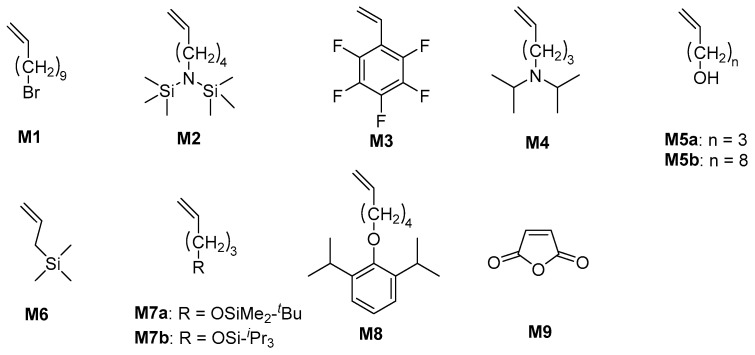
Polar comonomers for preparing PMP-based functionalized polymers.

Copolymerizing 4 MP with polar monomers to synthesize functional materials proves to be an efficient way of enhancing PMP’s performance. Zhang and coworkers synthesized PMP-based bromoalkyl functionalized polyolefins via copolymerization of 4 MP and 11-bromo-1-undecene (**M1**) using the Z-N catalyst (Figure 7) [101]. Subsequently, the PMP-Br polymers were quaternized by N(CH_3_)_3_, resulting in highly anion-conductive PMP-based electrolyte membranes for fuel cell applications. The highest ionic conductivity value (43 mS·cm^−1^) was achieved while maintaining low water uptake (29.2 wt%) at room temperature. They also synthesized PMP-based ionomers by copolymerization of 4 MP and bis (trimethylsilyl) amino-1-hexene (**M2**) [102]. Compared with pure PMP, the PMP-based ionomer membranes show a significantly improved dielectric constant (~5), higher breakdown field (612 MV·m^−1^), and good frequency and temperature stabilities (up to 160 °C). It can achieve a reliable energy storage capacity of over 7 J·cm^−3^, which is twice the energy storage capacity of state-of-the-art biaxially oriented polypropylene films. Kuehne synthesized copolymers of 4 MP and pentafluorostyrene (**M3**) using the Z-N catalyst, which has better solubility than PMP [103]. In addition, the critical surface tension γ_c_ of the copolymer is 25 mN·m^−1^, enabling electrospinning into non-wovens with superhydrophobic properties.

Waymouth reported the copolymerization of 4 MP with 5-N,N-diisopropylamino-1-pentene (**M4**) using metallocene catalysts [104]. The copolymers have higher decomposition temperatures with increasing amounts of polar monomers and can be protonated with HCl to yield a methanol-soluble material. Wang reported the copolymerization of 4 MP with α,ω-alkenol 9-decen-1-ol (9D1O, **M5a**) or 4-penten-1-ol (4P1O, **M5b**) [105] and allyltrimethylisilane (**M6**) [106] by using metallocene catalysts. 9D1O can significantly decrease the T_m_ of the copolymer, while 4P1O has no such great effect; even at an incorporation of 15.0 mol% for 4P1O, the T_m_ of the copolymer remains at a high level (T_m_ = 185 °C). The similar chain length of 4P1O enables it to be included in the crystallizable segment of 4 MP units. Wide-angle X-ray diffraction indicates that the incorporation of α,ω-alkenol can lead to a transition of polymer crystallization from Form II to I. The preparation of modified PMP materials using silicon-containing comonomers can effectively improve their wettability and biocompatibility.

Kisun’ko prepared linear isotactic copolymers of 4 MP with 5-(trialkylsiloxy)-1-pentene (**M7a** and **M7b**) using a zirconium post-metallocene catalyst [107]. The copolymers then reacted with Bu_4_NF to remove the siloxane groups, ultimately obtaining functional PMP with hydroxyl groups on the side chains. Recently, Jian synthesized polar functionalized ultra-high-molecular-weight PMP using pyridylamido hafnium catalysts through copolymerization of 4 MP and a polar monomer (**M8**). PMP-based functionalized polymers have a high isotacticity ([mmmm] up to 99%) and melting temperature (T_m_ > 205 °C) [55]. Additionally, Möller synthesized amphiphilic copolymers with strong antimicrobial properties mimicking natural antimicrobial peptides via free-radical polymerization using maleic anhydride (**M9**) and 4 MP as comonomers [108].

Although functional PMP materials show improved properties, industrial production of functional PMP materials remains a great challenge. The synthesis of functional PMP copolymers generated from copolymerization of 4 MP and polar monomers still shows low efficiency because polar comonomers often poison catalysts and decrease catalytic activity.

## 7. Conclusions and Outlook

Poly(4-methyl-1-pentene) (PMP) is a high-performance polyolefin resin synthesized using a variety of catalytic systems, including Ziegler–Natta, metallocene, post-metallocene, and late-transition metal catalysts. Notably, Ziegler–Natta and post-metallocene catalysts are particularly effective in producing highly isotactic PMP due to their ability to control the stereochemistry of polymers. The unique helical structure of PMP, combined with its large methyl side chains, results in a polymer with exceptional properties, such as low density, low dielectric constant, excellent chemical resistance, high transparency, and remarkable gas permeability. These attributes make PMP indispensable in the fields of electronics, medical devices, mobile communication, and renewable energy. PMP holds significant potential for further development. Blending PMP with nanomaterials or fiber reinforcements can enhance its mechanical properties, thereby expanding its applications in high-performance sectors such as the aerospace, automotive, and construction sectors. Additionally, the incorporation of functional additives, such as antibacterial agents, flame retardants, and conductive fillers, can yield innovative materials with specialized functionalities. Furthermore, copolymerization or blending PMP with polar monomers offers a pathway to create multifunctional materials, enhancing the compatibility and biodegradability of PMP, thus opening new opportunities in environmentally sustainable applications. Currently, PMP’s industrial production is not large, and its price is expensive, which limits its broader application. For scaling up PMP production, increasing the production of 4 MP monomer is also required. In general, the development of the production technique of 4 MP monomers as well as more efficient PMP catalysts and advanced polymerization technology would promote the entire PMP industrial chain.

## Figures and Tables

**Table 1:** Dielectric properties of different polymer materials.

Dielectric Property		PMP	PTFE	ETFE	PE
Dielectric constant	10 kHz	2.1	2.1	2.6	2.3
	1 MHz	2.1	2.1	2.6	2.3
	10 GHz	2.1	2.1	2.6	2.3
Dielectric dissipation	10 kHz	<0.0003	<0.0003	0.0006	-
	1 MHz	<0.0003	<0.0003	0.0015	-
	10 GHz	0.0008	0.0005	0.015	-

**Table 2:** Chemical resistance of different polymer materials.

Chemicals	PMP	PMMA	PC	PS	PA
Sulfuric acid (98%)	1	3	3	1	4
Sodium hydroxide (40%)	1	1	3	1	1
Ammonia water	1	1	3	1	1
Acetone	1	3	3	3	2
Methyl ethyl ketone	1	3	3	3	3
Ethanol	1	3	1	1	1
Toluene	3	5	3	5	-
Trichloroethylene	3	5	5	5	-

[25 °C], 1: not attacked, 2: practically not attacked, 3: attacked (swelling), 4: attacked (cracked), and 5: attacked (dissolve).

**Table 3:** Gas permeability of different polymer materials.

Permeability (10^−16^ mol·m/(m^2^·s·Pa)) [23 °C]				
Gas	PMP	HDPE	PP	PET
O_2_	94	0.06	5.17	0.04
N_2_	23.3	0.02	0.80	-
CO_2_	329	11.8	14.6	-

## References

[1] Wu R., Wu W.K., Stieglitz L., Gaan S., Rieger B., Heuberger M. (2023). Recent advances on α-diimine Ni and Pd complexes for catalyzed ethylene (Co) polymerization: A comprehensive review. Coord. Chem. Rev..

[2] Hustad P.D. (2009). Frontiers in olefin polymerization: Reinventing the world’s most common synthetic polymers. Science.

[3] Heggs T.G. (2011). Poly(4-methyl-1-pentene). Ullmann’s Encyclopedia of Industrial Chemistry.

[4] Kusanagi H., Takase M., Chatani Y., Tadokoro H. (1978). Crystal structure of isotactic poly(4-methyl-1-pentene). J. Polym.Sci. Polym. Phys..

[5] Evseev A.K., Zhuravel S.V., Alentiev A.Y., Goroncharovskaya I.V., Petrikov S.S. (2019). Membranes in extracorporeal blood oxygenation technology. Membr. Membr. Technol..

[6] Wu F., Lin Y., Wang L., Tang Y., Feng A., Yu L., Wang H., Wang X. (2024). Poly(4-methyl-1-pentene) hollow-fiber membranes with high plasma-leakage resistance prepared via thermally induced phase separation method. J. Membr. Sci..

[7] Aljishi R.S., Alkuaibi A.H., Al Zayer F.A., Al Matouq A.H. (2022). Extracorporeal membrane oxygenation for COVID-19: A systematic review. Cureus.

[8] Suh J., White J.L. (2007). Comparative study on quiescent crystallization kinetics of isotactic polyolefins. J. Appl. Polym. Sci..

[9] Charlet G., Delmas G.J.P. (1984). Effect of solvent on the polymorphism of poly(4-methylpentene-1): 2. Crystallization in semi-dilute solutions. Polymer.

[10] Daniel C., Vitillo J.G., Fasano G., Guerra G. (2011). Aerogels and polymorphism of isotactic poly(4-methyl-pentene-1). ACS Appl. Mater. Interfaces.

[11] Ruan J., Thierry A., Lotz B. (2006). A low symmetry structure of isotactic poly(4-methyl-pentene-1), Form II. An illustration of the impact of chain folding on polymer crystal structure and unit-cell symmetry. Polymer.

[12] De Rosa C. (2003). Crystal structure of Form II of isotactic poly(4-methyl-1-pentene). Macromolecules.

[13] Furushima Y., Masuda A., Kuroda T., Okada K., Iwata N., Ohkura M., Yamaguchi M. (2019). The effect of poly (4-methyl-1-pentene) on the nonisothermal crystallization kinetics of polypropylene. Polym. Cryst..

[14] Fu Y.J., Lai C.L., Hu C.C., Sun Y.M., Wu S.Y., Chen J.T., Huang S.H., Hung W.S., Lee K.R. (2016). Extraordinary transport behavior of gases in isothermally annealed poly(4-methyl-1-pentene) membranes. J. Polym. Sci. Pol. Phys..

[15] Mita K., Okumura H., Kimura K., Isaki T., Takenaka M., Kanaya T. (2013). Simultaneous small-and wide-angle X-ray scattering studies on the crystallization dynamics of poly(4-methylpentene-1) from melt. Polym. J..

[16] Chiba A. (2014). Structurally different amorphous solids of isotactic poly(4-methyl-1-pentene) and an apparently press-working-induced amorphization. J. Mol. Liq..

[17] Patil N., Invigorito C., Gahleitner M., Rastogi S. (2013). Influence of a particulate nucleating agent on the quiescent and flow-induced crystallization of isotactic polypropylene. Polymer.

[18] Wilsens C.H., Hawke L.G.D., Troisi E.M., Hermida-Merino D., de Kort G., Leoné N., Saralidze K., Peters G.W.M., Rastogi S. (2018). Effect of self-assembly of oxalamide based organic compounds on melt behavior, nucleation, and crystallization of isotactic polypropylene. Macromolecules.

[19] Markova S.Y., Gries T., Teplyakov V.V. (2020). Poly(4-methyl-1-pentene) as a semicrystalline polymeric matrix for gas separating membranes. J. Membr. Sci..

[20] Zhang T.Q., Hao S., Xiao J., Jia Z.Q. (2023). Preparation of poly(4-methyl-1-pentene) membranes by low-temperature thermally induced phase separation. ACS Appl. Polym. Mater..

[21] Han C., Lyu D., Lu Y., Men Y. (2023). Crystallinity and temperature dependent mechanical properties of poly (4-methyl-1-pentene). Polymer.

[22] Natta G., Pino P., Mazzanti G., Corrandini P., Giannini U. (1955). Synthesis and properties of crystalline high polymers of branched α-olefines. Stereoregular Polymers and Stereospecific Polymerizations.

[23] Kashiwa N., Yoshitake J. (1984). The influence of the valence state of titanium in MgCl_2_-supported titanium catalysts on olefin polymerization. Makromol. Chem..

[24] Kashiwa N., Yoshitake J. (1984). Polymerizations of α-olefins and styrene with MgCl_2_-supported titanium catalyst system: MgCl_2_/TiCl_4_/PhCO_2_Et with AlEt_3_PhCO_2_Et. Polym. Bull..

[25] Kashiwa N., Fukui K. (1987). Process for Production of 4-methyl-1-pentene Polymer or Copolymer. U.S. Patent.

[26] Altomare A., Solaro R., Ciardelli F., Barazzoni L., Menconi F., Masi F. (1992). Structural features of 4-methyl-1-pentene polymers prepared with different transition metal catalysts. Makromol. Chem. Macromol. Symp..

[27] Frolov I., Kleiner V., Krentsel B., Mardanov R., Munshi K., Bukatov G., Zakharov V., Sergeev S. (1993). Effect of the external donors on the polymerization of 4-methyl-1-pentene with high activity MgCl_2_/TiCl_4_ catalytic system. Makromol. Chem..

[28] Kissin Y.V. (2005). Structures of copolymers of high olefins. Fortschritte der Hochpolymeren-Forschung.

[29] Campbell T.W. (1961). Poly(4-methyl-1-pentene) and some soluble crystalline copolymers. J. Appl. Polym. Sci..

[30] Jones A.T. (1965). Cocrystallization in copolymers of α-olefins I—Copolymers of 4-methylpentene with linear α-olefins. Polym. Bull..

[31] Mizuno A., Yoshitake J.I., Muranaka T., Kitani H., Kashiwa N. (1992). A study of the structure and crystallinity of 4-methyl-1-pentene copolymers with 1, 5-hexadiene by ^13^C nuclear magnetic resonance and X-ray diffraction methods. Polymer.

[32] Kissin Y.V., Goldfarb Y.Y., Krentsel B., Uyliem H. (1972). Copolymerization of 4-methylpentene-1 and styrene with TiCl_3_ and ^i^Bu_3_Al and the copolymer structure. Eur. Polym. J..

[33] Kawahara N., Kojoh S., Matsuo S., Kaneko H., Matsugi T., Kashiwa N., Funaya M., Hirota N., Tanaka T., Hirose T. (2007). Olefin Polymer and Use Thereof. Patent.

[34] Wentz C.M., Fischbach D.M., Sita L.R. (2022). Stereomodulation of poly (4-methyl-1-pentene): Adoption of a neglected and misunderstood commercial polyolefin. Angew. Chem. Int. Ed..

[35] Xu G., Cheng D. (2001). Homo- and copolymerization of 4-methyl-1-pentene and ethylene with group 4 ansa-cyclopentadienylamido complexes. Macromolecules.

[36] Descour C., Duchateau R., Mosia M.R., Gruter G.J.M., Severn J.R., Rastogi S. (2011). Catalyst behaviour for 1-pentene and 4-methyl-1-pentene polymerisation for *C*_2_-, *C_s_*- and *C*_1_-symmetric zirconocenes. Polym. Chem..

[37] Pragliola S., Botta A., Longo P. (2017). Polymerization mechanism study of poly(4-methyl-1,3-pentadiene) and poly(4-methyl-1-pentene) prepared by using rac-[CH_2_(3-tert-butyl-1-indenyl)_2_]ZrCl_2_/^13^C enriched methylaluminoxane. Eur. Polym. J..

[38] Fischbach D.M., Wentz C.M., Mehdiabadi S., Soares J., Sita L.R. (2024). Stereoblock *vs* stereoblend: Orchestrating competing living coordination chain transfer polymerizations for the one-pot production of new viscoelastic grades of poly(4-methyl-1-pentene). ACS Catal..

[39] Losio S., Boccia A.C., Sacchi M.C. (2008). Ethylene/4-methyl-1-pentene copolymers by a “constrained geometry catalyst”: Advances in ^13^C NMR assignment. Macromol. Chem. Phys..

[40] Losio S., Stagnaro P., Motta T., Sacchi M.C., Piemontesi F., Galimberti M. (2008). Penultimate-unit effect in ethene/4-methyl-1-pentene copolymerization for a “sequential” distribution of comonomers. Macromolecules.

[41] Losio S., Tritto I., Zannoni G., Sacchi M.C. (2006). Ethene/4-methyl-1-pentene copolymers by metallocene-based catalysts:  An insight in ^13^C NMR assignment. Macromolecules.

[42] Stagnaro P., Boragno L., Losio S., Canetti M., Alfonso G.C., Galimberti M., Piemontesi F., Sacchi M.C. (2011). Isoselectivity and steric hindrance of *C*_2_ symmetric metallocenes as the keys to control structural and thermal features of ethene/4-methyl-1-pentene copolymers. Macromolecules.

[43] Galimberti M., Piemontesi F., Alagia L., Losio S., Boragno L., Stagnaro P., Sacchi M.C. (2010). Toward block copolymers from nonliving isospecific single-site catalytic systems. J. Polym. Sci. Part A Polym. Chem..

[44] Losio S., Forlini F., Boccia A.C., Sacchi M.C. (2011). Propene/4-methyl-1-pentene copolymers by metallocene-based catalysts: First insight into ^13^C NMR assignment. Macromolecules.

[45] Sacchi M.C., Losio S., Fantauzzi L., Stagnaro P., Utzeri R., Galimberti M. (2015). Random propene/4-methyl-1-pentene copolymers synthesized with *C*_2_ symmetric highly isospecific metallocenes. J. Polym. Sci. Part A Polym. Chem..

[46] Koval’chuk A.A., Klyamkina A.N., Aladyshev A.M., Nedorezova P.M., Antipov E.M. (2005). Copolymerization of propylene with 1-hexene and 4-methyl-1-pentene in liquid propylene medium. Synthesis and characterization of random metallocene copolymers with isotactic propylene sequences. Polym. Bull..

[47] Okamoto M., Mita K., Uekusa T., Takenaka M., Shibayama M. (2020). Development of elastic recovering 4-methyl-1-pentene/propylene copolymer. Polymer.

[48] Descour C., Meijer-Vissers T., Macko T., Parkinson M., Cavallo D., van Drongelen M., Hubner G., Goossens H., Duchateau R. (2012). Random and block copolymers based on 4-methyl-1-pentene and 1-pentene. Polymer.

[49] Toda T., Nakata N., Matsuo T., Ishii A. (2015). Extremely active α-olefin polymerization and copolymerization with ethylene catalyzed by a dMAO-activated zirconium(iv) dichloro complex having an [OSSO]-type ligand. RSC Adv..

[50] Nakata N., Toda T., Matsuo T., Ishii A. (2013). Controlled isospecific polymerization of α-olefins by hafnium complex incorporating with a trans-cyclooctanediyl-bridged [OSSO]-type bis(phenolate) ligand. Macromolecules.

[51] Boussie T.R., Diamond G.M., Goh C., Hall K.A., LaPointe A.M., Leclerc M., Lund C., Murphy V., Shoemaker J.A., Tracht U. (2003). A fully integrated high-throughput screening methodology for the discovery of new polyolefin catalysts: Discovery of a new class of high temperature single-site group (IV) copolymerization catalysts. J. Am. Chem. Soc..

[52] Wang L., Li D., Ren H., Wang Y., Wu W., Gao Y., Wang X., Gao H. (2021). Isoselective 4-methylpentene polymerization by pyridylamido hafnium catalysts. Polym. Chem..

[53] Wang L., Ni X., Ren H., Gao Y., Gao H. (2021). Homo- and copolymerization of 4-methyl-1-pentene and 1-hexene with pyridylamido hafnium catalyst. Acta Polym. Sin..

[54] Zheng L., Liu L., Shao C., Wang W., Wang B., Pan L., Li Y., Ma Z. (2019). Phase transition from tetragonal form II to hexagonal form I of butene-1/4-methyl-1-pentene random copolymers: Molecular factor versus stretching stimuli. Macromolecules.

[55] Zhou G., Mu H., Jian Z. (2024). Accessing functionalized ultra-high molecular weight poly(α-olefin)s via hafnium-mediated highly isospecific copolymerization. Macromol. Rapid Commun..

[56] De Stefano F., Scoti M., De Rosa C., Di Girolamo R.J.M. (2024). Synthesis and characterization of 4-methyl-1-pentene/1, 5-hexadiene isotactic copolymers with enhanced low-temperature mechanical performance. Macromolecules.

[57] Zheng H., Gao H. (2024). Noncovalent interactions in late transition metal-catalyzed polymerization of olefins. Macromolecules.

[58] Medina J.T., Tran Q.H., Hughes R.P., Wang X., Brookhart M., Daugulis O. (2024). Ethylene polymerizations catalyzed by fluorinated “sandwich” diimine-nickel and palladium complexes. J. Am. Chem. Soc..

[59] Zheng H., Qiu Z., Gao H., Li D., Cheng Z., Tu G., Gao H. (2024). Noncovalent Ni-phenyl interactions promoted α-diimine nickel-catalyzed copolymerization of ethylene and methyl acrylate. Macromolecules.

[60] Qiu Z., Wang W., Zheng H., Wang D., Zhao X., Tu G., Yang J., Gao H. (2024). Late transition metal olefin polymerization catalysts derived from 8-arylnaphthylamines. Inorganics.

[61] Gao H., Pan J., Guo L., Xiao D., Wu Q. (2011). Polymerization of 4-methyl-1-pentene catalyzed by α-diimine nickel catalysts: Living/controlled behavior, branch structure, and mechanism. Polymer.

[62] Gao H., Liu X., Tang Y., Pan J., Wu Q. (2011). Living/controlled polymerization of 4-methyl-1-pentene with α-diimine nickel-diethylaluminium chloride: Effect of alkylaluminium cocatalysts. Polym. Chem..

[63] Guo L., Gao H., Li L., Wu Q. (2011). Complex branched polyolefin generated from quasi-living polymerization of 4-methyl-1-pentene catalyzed by α-diimine palladium catalyst. Macromol. Chem. Phys..

[64] Liu J., Chen D., Wu H., Xiao Z., Gao H., Zhu F., Wu Q. (2014). Polymerization of α-olefins using a camphyl α-diimine nickel catalyst at elevated temperature. Macromolecules.

[65] Losio S., Leone G., Bertini F., Ricci G., Sacchi M.C., Boccia A.C. (2014). Ethylene–4-methyl-1-pentene copolymers of complex chain architecture using α-diimine Ni(II) catalysts: Synthesis, ^13^C NMR assignment and understanding the chain-walking mechanism. Polym. Chem..

[66] Leone G., Losio S., Piovani D., Sommazzi A., Ricci G. (2012). Living copolymerization of ethylene with 4-methyl-1-pentene by an α-diimine Ni(II)/Et_2_AlCl catalyst: Synthesis of diblock copolymers via sequential monomer addition. Polym. Chem..

[67] Ghule B., Laad M., Tiwari A.K. (2021). Poly-4-methyl-1-pentene a dielectric material: Patent landscape. J. Energy Storage.

[68] Gupta S., Offenbach I., Ronzello J., Cao Y., Boggs S., Weiss R.A., Cakmak M. (2017). Evaluation of poly (4-methyl-1-pentene) as a dielectric capacitor film for high-temperature energy storage applications. J. Polym. Sci. B Polym. Phys..

[69] Puleo A., Paul D., Wong P. (1989). Gas sorption and transport in semicrystalline poly (4-methyl-1-pentene). Polymer.

[70] Khoshbin E., Roberts N., Harvey C., Machin D., Killer H., Peek G.J., Sosnowski A.W., Firmin R.K. (2005). Poly-methyl pentene oxygenators have improved gas exchange capability and reduced transfusion requirements in adult extracorporeal membrane oxygenation. ASAIO J..

[71] Guo Y., Shao L., Zhang R., Gao W., Yu S., Du Y., Yang G., Pan F., Li T., Jiang Z. (2023). Modified poly(4-methyl-1-pentene) membranes by surface segregation for blood oxygenation. J. Membr. Sci..

[72] Ramanathan K., Antognini D., Combes A., Paden M., Zakhary B., Ogino M., MacLaren G., Brodie D., Shekar K. (2020). Planning and provision of ECMO services for severe ARDS during the COVID-19 pandemic and other outbreaks of emerging infectious diseases. Lancet Respir. Med..

[73] Markova S.Y., Pelzer M., Shalygin M.G., Vad T., Gries T., Teplyakov V.V. (2021). Gas separating hollow fibres from poly(4-methyl-1-pentene): A new development. Sep. Purif. Technol..

[74] Sheikh M., Lopez J., Reig M., Vecino X., Rezakazemi M., Valderrama C.A., Cortina J.L. (2023). Ammonia recovery from municipal wastewater using hybrid NaOH closed-loop membrane contactor and ion exchange system. Chem. Eng. J..

[75] Van de Witte P., Esselbrugge H., Peters A., Dijkstra P.J., Feijen J., Groenewegen R., Smid J., Olijslager J., Schakenraad J., Eenink M. (1993). Formation of porous membranes for drug delivery systems. J. Control. Release.

[76] Shin S.C., Yoon M.K. (2002). Application of TPX polymer membranes for the controlled release of triprolidine. Int. J. Pharm..

[77] Sønstevold L., Koza P., Czerkies M., Andreassen E., McMahon P., Vereshchagina E. (2024). Prototyping in polymethylpentene to enable oxygen-permeable on-a-chip cell culture and organ-on-a-chip devices suitable for microscopy. Micromachines.

[78] Zhong X., He T., Wang Z., Wang Y., Li L., Cui Z. (2023). Effect of β-cyclodextrin on the hemocompatibility of heparin-modified PMP hollow fibrous membrane for Extracorporeal Membrane Oxygenation (ECMO). Med. Nov. Technol. Devices.

[79] Lai A., Demarest C.T., Do-Nguyen C.C., Ukita R., Skoog D.J., Carleton N.M., Amoako K.A., Montoya P.J., Cook K.E. (2019). 72-Hour in vivo evaluation of nitric oxide generating artificial lung gas exchange fibers in sheep. Acta Mater..

[80] Arazawa D.T., Oh H.I., Ye S.H., Johnson C.A., Woolley J.R., Wagner W.R., Federspiel W.J. (2012). Immobilized carbonic anhydrase on hollow fiber membranes accelerates CO_2_ removal from blood. J. Membr. Sci..

[81] Pflaum M., Kühn-Kauffeldt M., Schmeckebier S., Dipresa D., Chauhan K., Wiegmann B., Haug R.J., Schein J., Haverich A., Korossis S. (2017). Endothelialization and characterization of titanium dioxide-coated gas-exchange membranes for application in the bioartificial lung. Acta Biomater..

[82] Malkin A.D., Ye S.H., Lee E.J., Yang X., Zhu Y., Gamble L.J., Federspiel W.J., Wagner W.R. (2018). Development of zwitterionic sulfobetaine block copolymer conjugation strategies for reduced platelet deposition in respiratory assist devices. J. Biomed. Mater. Res. Part B.

[83] Wiegmann B., von Seggern H., Höffler K., Korossis S., Dipresa D., Pflaum M., Schmeckebier S., Seume J., Haverich A. (2016). Developing a biohybrid lung-sufficient endothelialization of poly-4-methly-1-pentene gas exchange hollow-fiber membranes. J. Mech. Behav. Biomed. Mater..

[84] Habumugisha J.C., Usha Z.R., Yu R., Babiker D.M., Wan C., Chen X., Li L. (2021). Thermally stable and high electrochemical performance ultra-high molecular weight polyethylene/poly (4-methyl-1-pentene) blend film used as Li-ion battery separator. Appl. Mater. Today.

[85] Merkel K., Lenża J., Rydarowski H., Pawlak A., Wrzalik R. (2017). Characterization of structure and properties of polymer films made from blends of polyethylene with poly (4-methyl-1-pentene). J. Mater. Res..

[86] Gokkurt T., Gokkurt Y. (2024). Investigation of physical, thermal, mechanical and gas permeability properties of low-density polyethylene/poly (4-methyl-1-pentene) blend films and their utilization for fresh-cut fruit and vegetable packaging applications. Polym. Eng. Sci..

[87] Bu H., Nie G., Rong J. (2015). Crystallization and compatibility of poly (4-methyl-1-pentene) and polypropylene in their blends. J. Thermoplast. Compos. Mater..

[88] Lee K.H., Givens S.R., Snively C.M., Chase B., Rabolt J.F. (2008). Crystallization behavior of electrospun PB/PMP blend fibrous membranes. Macromolecules.

[89] Ilyin S., Ignatenko V., Anokhina T., Bakhtin D., Kostyuk A., Dmitrieva E., Antonov S., Volkov A. (2020). Formation of microfiltration membranes from PMP/PIB blends: Effect of PIB molecular weight on membrane properties. Membranes.

[90] Ignatenko V.Y., Anokhina T.S., Ilyin S.O., Kostyuk A.V., Bakhtin D.S., Antonov S.V., Volkov A.V. (2020). Fabrication of microfiltration membranes from polyisobutylene/polymethylpentene blends. Polym. Int..

[91] Feay D.C. (1985). Blends of Polycarbonate or Polyestercarbonate with 4-methyl-1-pentene Polymers. U.S. Patent.

[92] Xu L.Y., Ma A.P., Yin B., Yang M.B. (2019). The effect of alkylated graphene oxide on the crystal structure of poly(4-methyl-1-pentene) during uniaxial deformation at high temperature. Polym. Compos..

[93] Xu L.Y., Yan H.W., Gong L., Yin B., Yang M.B. (2015). Poly(4-methyl-1-pentene)/alkylated graphene oxide nanocomposites: The emergence of a new crystal structure. RSC Adv..

[94] Dorigato A., Pegoretti A. (2010). Tensile creep behaviour of polymethylpentene–silica nanocomposites. Polym. Int..

[95] Zainuddin M.I.F., Ahmad A.L., Subramaniam R.R. (2023). Poly(4-methyl-1-pentene)(PMP) with nano fumed silica as filler for CO_2_/N_2_ gas separation. Arab. J. Sci. Eng..

[96] Ghule B., Laad M. (2023). A study of solubility parameters on dispersion, dissolution, and homogenization of reinforcement TiO_2_ and poly(4-methyl-1-pentene) in different solvents for the fabrication of TiO_2_/PMP composite film. Chem. Eng. Commun..

[97] Choi Y.T., Kim S.B., Lee S.J., Kim G.T., Park E.H., Park E.S. (2017). Morphology, thermal, mechanical and electrical insulation properties of poly(4-methyl-1-pentene)/poly(ethylene-co-vinyl alcohol)-coated TiO_2_ nanocomposites. Compos. Part B Eng..

[98] Wanjale S.D., Jog J.P. (2003). Effect of modified layered silicates and compatibilizer on properties of PMP/clay nanocomposites. J. Appl. Polym. Sci..

[99] Wanjale S.D., Jog J.P. (2004). Poly(4-methyl-1-pentene)/clay nanocomposites: Effect of organically modified layered silicates. Polym. Int..

[100] Narejo A.R., Qureshi R.F., Almas R., Memon S.I., Mahar F.K., Syed N., Ahmed F., Khatri Z. (2022). Fabrication and characterization of co-electrospun cellulose/poly (4-methyl-1-pentene) nanofibers with improved tensile properties. Mater. Res. Express.

[101] Zhang M., Shan C., Liu L., Liao J., Chen Q., Zhu M., Wang Y., An L., Li N. (2016). Facilitating anion transport in polyolefin-based anion exchange membranes via bulky side chains. ACS Appl. Mater. Interfaces.

[102] Zhang M., Zhang L., Zhu M., Wang Y., Li N., Zhang Z., Chen Q., An L., Lin Y., Nan C. (2016). Controlled functionalization of poly(4-methyl-1-pentene) films for high energy storage applications. J. Mater. Chem. A.

[103] Usselmann M., Hefty L., Welscher P.J., Kuehne A.J.C. (2023). One-step Ziegler-Natta polymerization of 4-methylpent-1-ene with pentafluorostyrene–A solution processable copolymer with super hydrophobic properties. Polymer.

[104] Stehling U.M., Stein K.M., Fischer D., Waymouth R.M. (1999). Metallocene/borate-catalyzed copolymerization of 5-N,N-diisopropylamino-1-pentene with 1-hexene or 4-methyl-1-pentene. Macromolecules.

[105] Wang W., Hou L., Sheng J., Ren M., Tang Y. (2016). Copolymerization of 4-methyl-1-pentene with α,ω-alkenols. Express Polym. Lett..

[106] Wang W., Ren M., Hou L., Qu S., Li X., Guo Z. (2023). Polymerization of allyltrimethylisilane and 4-methyl-1-pentene by using metallocene catalysts. Polymers.

[107] Kisun’ko D.A., Lemenovskii D.A., Aladyshev A.M. (2006). Synthesis of isotactic copolymers of 4-methyl-1-pentene by living polymerization catalyzed by zirconium non-metallocene complexes. Polym. Sci. Ser. A.

[108] Szkudlarek M., Heine E., Keul H., Beginn U., Möller M. (2018). Synthesis, characterization, and antimicrobial properties of peptides mimicking copolymers of maleic anhydride and 4-methyl-1-pentene. Int. J. Mol. Sci..

